# Supplementary material for: High-resolution genome assembly and linkage mapping in Meloidogyne hapla reveal non-canonical telomere repeats and recombination hotspots associated with effector proteins
Source: PLoS Pathog. 2025 Nov 18;21(11):e1013706. doi: 10.1371/journal.ppat.1013706 (PMC12643312; doi:10.1371/journal.ppat.1013706)
Supplement: Attachment — Submitted filename: Reviewer Response_Shakya et al., 2025.docx [file ppat.1013706.s029.docx]

**Dear Editor,**

Thank you for the opportunity to revise our manuscript submitted to PLOS Pathogens. We have carefully addressed all comments from the reviewers and made corresponding changes to the manuscript. A detailed point-by-point response is provided below, highlighting how each comment has been addressed.

We believe the revised manuscript has been significantly improved and now meets the journal’s standards. We sincerely hope it is now suitable for publication.

Best regards,

**Dr. Shahid Siddique**

**Reviewer #1:**

P16, L18 (6th line from the bottom): Change ‘virulence’ to ‘parasitism’
Response: This has been updated now. The sentence now reads “…parasitism…”.

 P18, L3: Remove the ‘- (hyphen)’ from ‘Simi-larly’
Response: Corrected. “Similarly” is now spelled correctly.

 P19, L11: Remove the repetitive words: ‘show noticeable’
Response: The redundant phrase has been removed.

 P24, 3rd line from the bottom: Remove the repetitive sentence: ‘This observation is further supported by FISH analysis, which detects these repeats at a single chromosome end.’
Response: The duplicate sentence has been deleted.

P42, L9: Change ‘O/N’ to ‘overnight’
Response: Revised accordingly. ‘O/N’ has been replaced with ‘overnight’.

**Reviewer #2: TANDEM REPEAT ELEMENTS**

My one significant scientific question is about the ascertainment of short tandemly repeated sequence elements. The authors observed a novel 16-mer forming tandem repeats at the ends of several chromosomes (as well as in the center of chromosomal scaffold 1). Given the way the text is written, they apparently observed *no* other short tandemly repeated elements at the ends of chromosomes (e.g., at either end of chromosomal scaffold 1, at the right end of chromosomes 3 and 4, at the left end of chromosome 6, etc.). It is not clear from the Methods whether the Tandem Repeats Finder (TRF) was used genomewide to identify possible tandem repeats, or whether it was run only in the terminal 24 kb of each chromosome. It is also not clear whether the authors did or did not observe any other significant tandemly repeated elements in any chromosomal end regions other than the 16-mer that they describe here (the reader is left to assume that the authors did not). The authors note in the Discussion that "we cannot rule out the possibility that ... additional relevant sequences remain undetected".

I would like the authors to do the following:

1. Explicitly state in their Results whether TRF did, or did not, identify any other tandemly repeated elements other than the 16-mer in the terminal 24 kb of chromosomal scaffolds. If TRF did identify such elements, add them to their existing Supp. Table 1.

I would also like the authors to strongly consider doing the following:

2. In addition to their existing analyses, use an alternative method to identify telomere-associated tandemly repeated elements directly from their long-read sequence data (which they have in abundance -- they have 56x genomic coverage of high-accuracy HiFi reads, as well as 143x coverage of less accurate Oxford Nanopore reads). One recently published method that the authors could use for this analysis is TeloSearchLR.

TeloSearchLR code:<https://github.com/gchchung/TeloSearchLR>

TeloSearchLR bioconda package:<https://bioconda.github.io/recipes/telosearchlr/README.html>

TeloSearchLR PubMed ID:<https://pubmed.ncbi.nlm.nih.gov/40169380>

TeloSearchLR paper:<https://academic.oup.com/g3journal/article/15/6/jkaf062/8102967>

By running TeloSearchLR, the authors should rediscover the 16-mer as a positive control result. But in addition, any other tandem repeats associated with telomeres (e.g., at either end of chromosomal scaffold 1) may also be discoverable by TeloSearchLR. Having such a result could make their analysis of the *Meloidogyne hapla* VW9 genome more complete.

Response: We thank the reviewer for these valuable suggestions and the opportunity to clarify and extend our analysis. We have revisited our analysis of telomere-associated repeats and performed additional validation steps, as detailed below.

**TRF Analysis**: We initially applied Tandem Repeat Finder (TRF) to the terminal 24 kb of each scaffold independently and identified a highly prevalent 16-mer tandem repeat at the ends of 17 scaffolds in 3’ direction. This 16-mer was the only significant repeat detected in terminal regions. We then inspected entire S1 region, and identified a similar albeit more degenerate repeat, in the center. A subsequent scan of entire scaffolds confirmed no additional distinct tandem repeats meeting our defined thresholds. Detailed methodology on this approach is included in the "Terminal repeat regions" section of our manuscript

**TeloSearchLR Validation**: Following the reviewer’s suggestion, we used TeloSearchLR on ultra-long ONT reads, analyzing both 1 kb and 5 kb terminal windows. This analysis confirmed the 16-mer repeat as the top-ranked motif. Other motifs identified were either variants of the 16-mer or non-terminal repeats not enriched at chromosome ends. These results further validate the 16-mer as the dominant telomere-associated repeat in *M. hapla* VW9.

**Additional Motifs (MEME)**: We also used MEME to explore scaffold ends. This information has been added to page 10-11. Two additional motifs were found: one at the ends of chromosomes 3, 10, 13, and 15, and another at ends of chromosomes 1 and 11. However, these do not yet have independent validation (e.g., FISH or long-read confirmation). We now also refer to these repeats in the Discussion.

**MINOR QUESTIONS AND SUGGESTED CORRECTIONS**

In the Introduction:

"Meloidogyne spp. exhibits" should read ""Meloidogyne spp. exhibit" ('spp.' abbreviates '[two or more] species', which will take a plural verb).

Response: This has been corrected.

In the Results:

"...with the consensus sequence (CCCAAGGTTAAAAGG) at 17 scaffold ends." This sentence and the paragraph it starts are not incorrect as they stand, but the way they were organized (and the fact that no mention at all of variant tandem repeats is made in the legend of Figure 1) initially confused me badly when I read this, and if left unchanged may confuse readers as well. To make things clearer, I would suggest revising the first sentence to read ".....with the consensus sequence (CCCAAGGTTAAAAGG) at 17 scaffold ends, and with one variant at an 18th scaffold end." The rest of the paragraph can then be left unchanged but readers should be less easily confused.

Response: We have now revised the sentence in the Results section to explicitly mention the presence of the variant repeat at one scaffold end. The updated sentence now reads:

“To define scaffold ends in the *M. hapla* assembly, we analyzed tandem repeats within the terminal 24 kb of each scaffold. This analysis revealed arrays of a 16-mer with the consensus sequence (CCCAAGGTTTAAAAGG) at 17 scaffold ends, and a variant repeat at one end of Scaffold 10.”

"at the center of S1 scaffold" should read "at the center of the S1 scaffold".
Response: Corrected as suggested.

Figure 1 shows the distribution of tandem repeats in all chromosomes, and *includes* the repeats at the left end of scaffold S10, but does not tell (or remind) the reader that S10's tandem repeats are of a variant repeat unit. An additional sentence should be added to the legend of Figure 1B to make this obvious. It might say: "Except for scaffold S10, all repeats have a 16-mer consensus element (shown); those in S10 have a variant element."

Response: Thank you for the suggestion. We have updated the legend to state: “The repeat at the end of Scaffold 10 is a variant of the others shown..”

"Of the 1258 genes encoding PSPs..." should (I think) read "Of the 1,258 genes encoding PSPs..."

Response: We have changed 1258 genes to 1,248 genes throughout the manuscript.

"p-value=2.5e-08" with the '08' in superscript could be better written either by not putting the '08' in superscript (which would at least make the text more readable) or by instead writing out the numbers as "p-value = 2.5•10-8".

Response: We have reformatted all p-values to improve readability. For example, P = 2.5 × 10⁻⁸.

Reviewer #3:

1) I find the mitochondrial genome assembly workflow to be a bit strange. The authors identified contig 351 as mitochondrial. However, they then assembled the mitochondrion de novo using only the Illumina reads with MitoZ. Why didn't the authors polish contig 351, or alternatively, why didn't they utilise the available long reads? Regarding the tRNA annotation, did the authors compare with the mitochondria of other Meloidogyne species to check how many tRNAs they have?

Response: We initially identified contig 351 as mitochondrial based on sequence similarity and coverage profiles. Following this, we tested several assembly strategies, including (i) polishing contig 351 with short reads and (ii) assembling the mitochondrial genome de novo using both Illumina and long-read (Oxford Nanopore and PacBio HiFi) data. However, these versions yielded incomplete or inconsistent annotation of mitochondrial genome. In contrast, a de novo Illumina‑based assembly generated with MitoZ, followed by annotation with MITOS2, yielded the most complete and consistent gene set. As such, we present this version as our reference. Additional methodological details have been now added in the Methods section Mitochondrial genome assembly and annotation.

Regarding tRNA annotation, standard pipelines including tRNAscan-SE were unable to detect the full set of expected mitochondrial tRNAs. To address this, we used the mitochondrial genome of the closely related species *Meloidogyne graminicola* as a reference to manually inspect and annotate potential tRNA loci. We suspect the remaining five are present but undetectable due to non-canonical tRNA structures (e.g., armless tRNAs), which are common in nematodes. This explanation has been added to the Methods section.

2) The Nigon analysis is not integrated into the overall flow of the article. I would assume that the genomic structural variability even between *M. hapla* strains (as shown in the next section) is responsible for the Fig. 3 result. Maybe the authors can add a few sentences to connect these two findings.

Response: We appreciate this comment. Figure 3 is intended to show conservation and divergence of ancestral linkage blocks (Nigon elements) across rhabditid nematodes. Our analysis of Nigon elements reflects evolutionary-scale chromosomal organization changes that likely predated *M. hapla* speciation rather than the more recent strain-level differences. To clarify this, we have added the following sentence to the Results:

“This pattern of Nigon elements suggests that extensive chromosomal rearrangements have occurred since *M. hapla* diverged from the ancestral 7-chromosome rhabditid species.”

3) There are multiple good-quality Meloidogyne assemblies available. Have the authors considered using them for comparative structural analyses?

Response: We agree that comparative structural analysis across high-quality *Meloidogyne* genomes provide powerful insights into evolutionary trajectories and genome architecture within the genus. While such analyses are indeed a priority for our ongoing research, they involve extensive orthology mapping, synteny comparisons, and structural alignment workflows that are beyond the current manuscript’s scope. Given the breadth of data generated in this study, we have chosen to focus on presenting the *M. hapla* VW9 genome in detail. We plan to include comparative genomic analyses with additional *Meloidogyne* species in a follow-up study and have now indicated this direction in the Discussion.

4) The Iso-Seq and RNA-seq data were used only for annotation purposes. Have the authors considered examining expression patterns - if any - and mapping them to the PSPs inside and outside of the HMZs?

Response: We thank the reviewer for this suggestion. While our current use of Iso-Seq and RNA-seq data is limited to improving structural gene annotation, we agree that examining expression patterns especially of PSPs located inside and outside of hypervariable (HMZ) regions would be highly informative. We are currently planning a follow-up analysis to investigate differential expression of PSPs under different life stages and environmental conditions, with a particular focus on their genomic context (e.g., HMZs vs. non-HMZs).

\# Typos (P=Page, L=Line)

P.18 - L. 3 : Simi-larly to Similarly

- L. 5 : Ta-ble to Table

Response: Corrected.

P.19 - L. 11 : "show noticeable" is written twice

Response: Corrected.

P.24 - : "a finding further supported by FISH analysis, which detects these repeats at a single chromosome end" is written twice

Response: Corrected.
